# “*How*” web searches change under stress

**DOI:** 10.1038/s41598-024-65895-4

**Published:** 2024-07-02

**Authors:** Christopher A. Kelly, Bastien Blain, Tali Sharot

**Affiliations:** 1 Department of Experimental Psychology, University College London, London, WC1H 0AP UK; 2 Max Planck University College London Centre for Computational Psychiatry and Ageing Research, London, WC1B 5EH UK; 3 Department of Brain and Cognitive Sciences, Massachusetts Institute of Technology, Cambridge, 02139 USA

**Keywords:** Psychology, Human behaviour

## Abstract

To adjust to stressful environments, people seek information. Here, we show that in response to stressful public and private events the high-level features of information people seek online alter, reflecting their motives for seeking knowledge. We first show that when people want information to guide action they selectively ask “*How*” questions. Next, we reveal that “*How*” searches submitted to Google increased dramatically during the pandemic (controlling for search volume). Strikingly, the proportion of these searches predicted weekly self-reported stress of ~ 17K individuals. To rule out third factors we manipulate stress and find that “*How*” searches increase in response to stressful, personal, events. The findings suggest that under stress people ask questions to guide action, and mental state is reflected in features that tap into why people seek information rather than the topics they search for. Tracking such features may provide clues regrading population stress levels.

## Introduction

Every person will face unexpected adversities during their lifetime. These stressful events may be global (e.g., war, pandemic) or unique to the individual (e.g., being diagnosed with cancer, losing one’s job, divorce). Such events often lead to stress, anxiety, confusion, and a reduced sense of control^1–6^.

One available adaptive reaction is to seek information that can help guide action to promote adaptation^7–9^. Such actions can be directly related to the event experienced (e.g., during wartime people may search for information on how to secure windows from being shuttered by rockets) or indirectly related (e.g., searching for information on activities that can distract oneself from the adversity).

To date, research on the relationship between information-seeking and stress has mostly focused on the frequency of information-seeking. Some studies propose that stress is associated with greater information seeking^10–12^, which may decrease the sense of uncertainty that is heightened under stress. Others, however, suggest that stress leads to an avoidance reaction which is characterised by less information-seeking about the stressor^13,14^.

Here, we take a different theoretical viewpoint. Rather than focusing on whether stress generally enhances or reduces information seeking, we test the hypothesis that when experiencing abrupt stressful life events people are more likely to search for information that can direct *action*—a reaction which may be adaptive. People may seek information that can direct action to deal with the stressful event directly (e.g., during a pandemic a person may ask “*how* *do* *I* *make* *a* *mask?*”) and/or information to direct action that distracts from the stressor (e.g., “*how* *do* *I* *make* *a* *Margarita?*”). In other words, stress may alter the type, rather than frequency, of information people seek. Across multiple studies, we test whether such changes can be detected and quantified by simple analysis of web searches. We propose that by examining the features of the information that people seek, we can gain insight into their external state and their internal reaction to that state.

To that end, we also examine whether the negative context people find themselves in is associated with a change in the valence of web searches. That is, whether a negative state may lead to more negatively valanced searches due to that state, or alternatively to more positive searches in an attempt perhaps to counter the negative state. The former possibility is supported by studies showing that anxious individuals have a bias towards negative stimuli^15–17^, which suggests that anxiety may increase the search for negative information.

We test our proposal both in the context of a global stressful event (i.e., the COVID-19 pandemic) and of personal aversive life events. Our approach differs dramatically from past attempts to relate web searches to mental state. Current research on web searches has focused on relating web searches for specific content terms (e.g., “*suicide*, “*anxiety*”, “*Prozac*”) with mental health indicators of a population, a method that has resulted in mixed findings^18–31^. In contrast, we assess whether changes in the *high-level* *features* of searches reflect stress levels. This approach, which is based on a recent theory of information-seeking motives^8^, may be more sensitive as it does not make an assumption about which topics people are searching for, but rather the characteristic of information they are searching for (i.e., information that may guide action).

First, we conducted a control study to identify which question-words people would use when seeking information to guide action (Study 1). The results clearly show that participants selectively use “*How*” for this purpose. Next in Study 2, we calculated the percentage of Google searches containing the “*How*” question-word submitted in the UK and US out of all searches submitted in that region, every week for a year from the date a “*National* *Emergency*” was declared and compared to the years before. Changes to the proportion of “*How*” searches cannot be explained by changes in the volume of Google searches during the pandemic, as we examined the change in the *percentage* of “*How*” searches out of all searches at that time. We also quantified the valence of the most frequent questions submitted to the Google search engine each week in the UK and US using a machine learning approach (HuggingFace, 2022). We then examined how these features related to weekly stress reports of approximately 17K individuals in the UK. Importantly, we dissociate the effects of stress on information-seeking from the effect of COVID-19 related confinement. Together, these analyses enable us to examine how features of web searches alter under a global stressor.

To be able to generalize our findings to private stressful events and to rule out potential third factors, we then tested our hypotheses in a controlled environment in Study 3. Here, we manipulate stress levels and examined if the manipulated stress impacts the likelihood of asking “*How*” questions in relation to private events.

## Results

### Study 1

#### “*How*” web searches are selectively associated with the need to guide action

To determine which question-words are associated with guiding action, we asked 100 participants (Age = 39.29 (SD = 13.86), Females = 56%, Males = 44%, Other = 0%) to think about a goal they were trying to achieve. They were then instructed to select from a list of eight question words (“*What*”, “*Which*”, “*Who*”, “*Where*”, “*Why*”, “*When*”, “*Whose*”, and “*How*”), the word that they would use in a Google query to help guide their actions to achieve the goal (i.e., test question). Participants were also asked to think about a topic they are interested in learning more about. They were then instructed to select a question-word from the same list of eight words, that they would use in Google query to increase their knowledge about the topic (i.e., control question). This design allowed us to identify which words people use to seek information to guide action and test whether such words were used generally for information-seeking, or more specifically to guide action.

**Figure 1:**
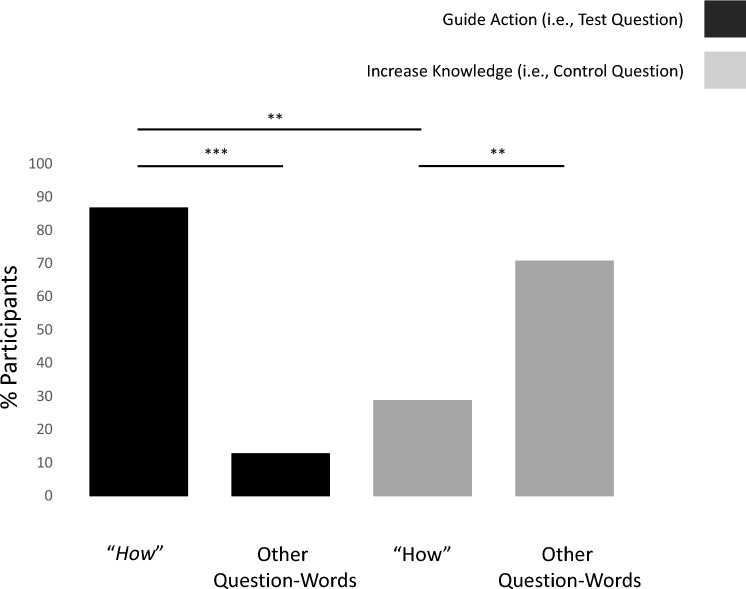
“*How*” questions are associated with guiding actions. Plotted on the y-axis is the percentage of participants selecting a particular question-word. Participants were more likely to select “*How*” over other question-words when asking a question to help guide their actions to achieve a goal. They also were more likely to ask “*How*” to help guide their actions than to simply increase their understanding. ***p < 0.001, **p < 0.01.

The likelihood of selecting the word “*How*” to ask a question to guide action was equal to 87%, which was significantly greater than the likelihood of selecting “*How*” to increase understanding, which was 29%. (p = 0.009, Fisher exact test see Fig. 1). Moreover, the likelihood of selecting “*How*” to guide action was significantly greater than the likelihood of selecting all other question words put together (p < 0.001, Fisher exact test; see Fig. 1), while for increasing knowledge, the likelihood of selecting “*How*” was significantly less than the likelihood of selecting all other question words put together (p < 0.001, Fisher exact test; see Fig. 1).

Given the results of Study 1, we used “*How*” questions in Study 2 and 3 as a proxy for the desire to gain knowledge that could help guide action. Note that we are not suggesting that all questions that begin with “*How*” are intended to guide action. Rather that, on average, if people want to ask a question to guide action, they will be likely to use “*How*”. In contrast, they are not especially likely to use “*How*” simply to learn more about a topic.

### Study 2

#### The pandemic resulted in a significant change to the high-level features of web searches

To assess the high-level feature of web searches associated with guiding action, motivated from Study 1, we extracted the Google Search Volume Index of “*How*” questions. A Google Search Volume Index is equal to the number of searches for the specific term of interest in a given week and region divided by the total number of searches for that same week and region. These percentages are then normalized to represent search interest relative to the highest percent for that region for the entire time frame (i.e., January 1st, 2017–March 21st, 2021; see Methods for details). Note, that weekly changes to the Google Search Volume Index cannot be explained by weekly changes in the total volume of Google searches, as the index reflects the *percent* of specific searches out of all searches that week (i.e., on a scale from 0 to 100). We also calculated a second feature—a Valence Index—which indicates the valence of the most frequent questions submitted to Google search engine. To calculate this index, we first extracted the most frequent web searches each week that included question-words (i.e., “*What*” “*Which*”, “*Who*”, “*Where*”, “*Why*”, “*When*”, “*Whose*”, and “*How*”). Next, we implemented a machine learning approach to assess the valence of searches, by applying the pre-trained model distilbert-base-uncased-finetuned-sst-2-english (HuggingFace, 2022) to the extracted data. The model’s output contains a label: positive and negative, along with a score between 1 and 0 (1 = absolute confidence in the model output label, 0 = zero confidence in the model output label). As the sum of both values is equal to 1, we used only the positive confidence score as our measurement of valence and then transformed this number to be on a scale from 0 to 100 (i.e., a score of 100 denotes the most positively valenced score and a score of 0 the most negatively valenced score), such that it would be easily comparable to the Google Search Volume Index for “*How*”, described above. We validated the algorithm score in this context by asking a naïve human subject to categorize 192 randomly sampled searches as either ‘more positive’ or ‘more negative’. We found a significant positive association between the algorithmic score and the human score (r(190) = 0.463, p < 0.001). Note, that this correlation is only of medium size, which may be because when humans rate the valence of a question they consider the predicted answer while the algorithm does not.

**Figure 2:**
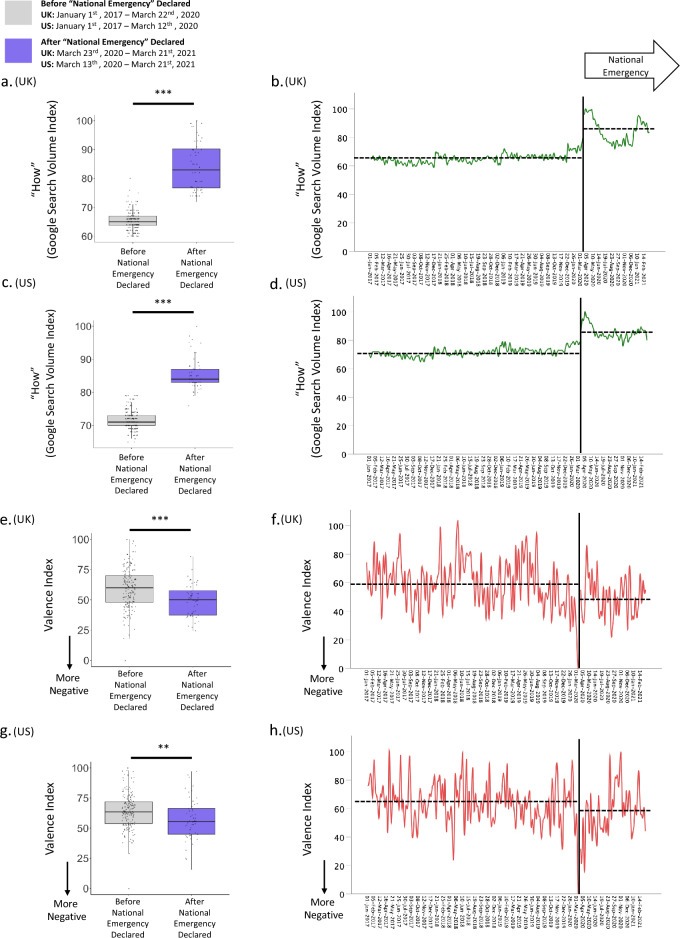
High-level characteristics of web searches alter during the pandemic. Relative volume of “*How*” searches (i.e., the proportion of “*How*” searches relative to all searches for that time and place) was greater after the COVID-19 “*National* *Emergency*” declaration relative to before in (**a**,**b**) the UK and (**c**,**d**) the US. The Valence Index [0 (most negative valenced) to 100 (most positive valenced)] reveals that question queries submitted to the Google search engine were more negatively valenced in the (**e**,**f**) UK and (**g**,**h**) the US after the COVID-19 “*National* *Emergency*” declaration relative to before. The period assessed prior to the “*National* *Emergency*” was from January 1st, 2017 to the declaration of each country’s “*National* *Emergency*”. The “*National* *Emergency*” was assessed from March 23rd, 2020 to March 21st, 2021, in the (**a**,**b**,**e**,**f**) UK and from Match 13th, 2020 to March 21st, 2021 in the (**c**,**d**,**g**,**h**) US. (**a**,**c**,**e**,**g**) The horizontal lines indicate median values, boxes indicate 25–75% interquartile range and whiskers indicate 1.5× interquartile range; individual scores are shown as dots. (**b**,**d**,**f**,**h**) The bold line indicates the declaration of the “*National* *Emergency*”, the dashed lines indicate the mean values for before and after the “*National* *Emergency*”. ***p < 0.001, **p < 0.01 (two-sided).

Analyses were conducted separately in the UK and the US. In each country we quantified the measures above for every week from the date the “*National* *Emergency*” was declared (UK: March 23rd, 2020; and US: March 13th, 2020) through March 21st, 2021, as well as every week from January 1st, 2017 until “*National* *Emergency*” was declared. There was a significant increase in the Google Search Volume Index of “*How*” questions following the declaration of a “*National* *Emergency*” relative to the three years previous (“*How*” UK: before “*National* *Emergency*” declared: M = 65.37, SD = 3.27, after “*National* *Emergency*” declared: M = 84.19, SD = 8.52, t(55.71) = − 15.585, p < 0.001, Cohen’s d = − 3.754; “*How*” US: before “*National* *Emergency*” declared: M = 71.65, SD = 2.87, after “*National* *Emergency*” declared: M = 85.62, SD = 4.90, t(63.67) = − 19.682, p < 0.001, Cohen’s d = − 4.029; Fig. 2a–d).

Moreover, the Valence Index for question queries after the “*National* *Emergency*” was declared was found to be more negative compared to before (Valence Index UK: before “*National* *Emergency*” declared: M = 59.37, SD = 16.71, after the “*National* *Emergency*” declared: M = 49.77, SD = 13.26, t(218) = 3.788, p < 0.001, Cohen’s d = 0.601; Valence Index US: before “*National* *Emergency*” declared: M = 64.09, SD = 14.52, after “*National* *Emergency*” declared: M = 57.07, SD = 17.41, t(218) = 2.917, p = 0.004, Cohen’s d = 0.460; Fig. 2e–h).

#### Population stress-levels are selectively associated with asking “*How*”

Thus far, we have shown that there is an increase in the proportion of “*How*” and negatively valenced searches submitted to the Google search engine during the pandemic relative to before. We next examined whether these were related to population stress levels. We had access to self-report stress levels collected every week in the UK between March 21st, 2020 and March 21st, 2021. Approximately 70K unique individuals completed the survey, on average 17,468 individuals a week in the UK^32^. Specifically, participants were asked to indicate if over the previous week they felt worried and/or stressed about any of the following factors: (i) catching COVID-19, (ii) becoming seriously ill from COVID-19, (iii) finance, (iv) unemployment and (v) getting food. We computed the mean proportion of individuals who reported stress or worry over these factors. We conducted separate linear models, each predicting on a weekly basis either the Google Search Volume Index in the UK of “*How*” searches or the Valence Index, from stress and from UK COVID-19 confinement scores. The inclusion of the latter enabled us to disentangle the effects of stress on web searches from the effects of confinement due to restrictions placed by the Government. COVID-19 related confinement data for each week in the UK was obtained from the Oxford University COVID-19 Government Response Tracker^33^. The data includes ordinal variables coded by severity/intensity of confinement, on a daily basis (from January 1st, 2020 to March 21st, 2021), due to the following: (i) school and university closures, (ii) workplace closures, (iii) public event cancelations, (iv) restrictions on gatherings, (v) public transport restrictions, (vi) stay at home requirements, (vii) restrictions on domestic travel, and (viii) restrictions on international travel; see Table 4 for coding. To obtain weekly values, we computed weekly averages of the daily ratings, where all eight variables were normalised to range between 0 and 1 and averaged together.

Importantly, to account for simple temporal trends we removed linear trend^34,35^ from the dependent variables and predictor variables (stress scores and COVID-19 related confinement), using the detrend function in the ‘pracma’ R package. The detrended dependent and predictor variables were then Z-scored before being entered in the linear models.

**Figure 3:**
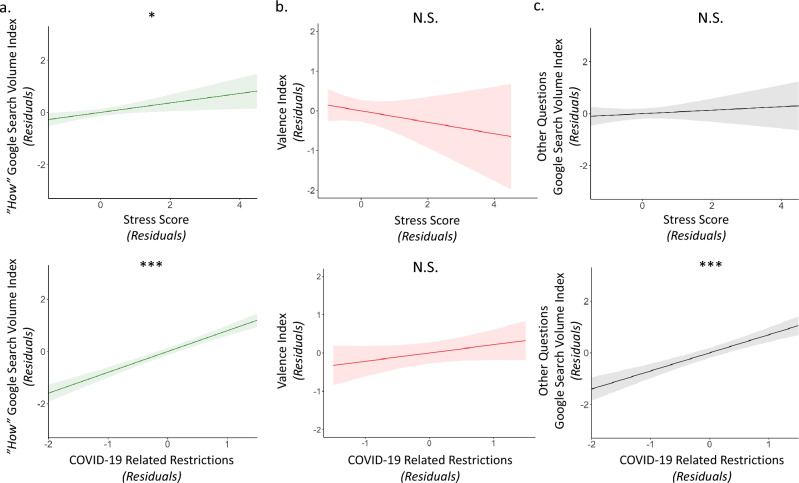
Self-reported stress is selectively associated with an increase in “*How*” searches. Stress level in the UK was associated with (**a**) the UK Google Search Volume Index of “*How*” searches (detrended and Z scored), but not with (**b**) the Valence Index [0 (most negative valenced) and 100 (most positive valenced)] (detrended and Z scored), nor with (**c**) the mean UK Google Search Volume Index of other questions (i.e., What, Which, Who, Where, Why, When, and Whose; detrended and Z scored). UK COVID-19 related confinement score (detrended and Z scored; bottom panel) was associated with both (**a**) the UK Google Search Volume Index of “*How*” searches and (**c**) the mean UK Google Search Volume Index of other questions, but not (**b**) the Valence Index. Stress levels and COVID-19 confinement scores (all detrended and Z-scored) were entered in the same models, controlling for each other. The X and Y values are the residuals (regressing out the respective control variable). The fine line represents the confidence interval. ***p < 0.001, *p < 0.05, *N.S.* not significant (two-sided).

The linear model predicting “*How*” questions from stress levels and COVID-19 related confinement scores, revealed that both high stress (β = 0.182 ± 0.074 (SE), t(49) = 2.464, p = 0.017) and greater COVID-19 related confinement (β = 0.797 ± 0.074 (SE), t(49) = 10.812, p < 0.001; Fig. 3a) predicted proportion of “*How*” searches. In other words, the relationship between stress levels and “*How*” searches cannot be solely explained by increased restrictions during the pandemic, as our model controls for COVID-19 related confinement.

We then conducted the same linear model as above, but this time predicting the Valence Index. The Valence of searches was not predicted by either variables (stress: β = − 0.144 ± 0.147 (SE), t(49) = − 0.980, p = 0.332, COVID-19 related confinement: β = 0.216 ± 0.147 (SE), t(49) = 1.470, p = 0.148; Fig. 3b).

Next, to examine if the relationship between stress and “*How*” searches was specific, or rather reflected a general tendency to ask more questions when stressed, we conducted a third linear model relating question asking to stress levels controlling for confinement. In particular, we extracted the Google Search Volume Index for all other common question-words (i.e., What, Which, Who, Where, Why, When, and Whose; Z-scored and detrended) and then averaged these together. We then predicted the average Google Search Volume Index of all other common question-words from stress scores and COVID-19 related confinement. Importantly, the proportion of other common questions asked was selectively predicted by COVID-19 related confinement (β = 0.701 ± 0.104 (SE), t(49) = 6.744, p < 0.0001), but not stress levels (β = 0.067 ± 0.104 (SE), t(49) = 0.645, p = 0.522; Fig. 3c).

Thus far, we have shown that the relative volume of searches that can direct action is related to stress levels. Next, we wanted to test the predictive validity of this simple model. Specifically, we used our stress score to predict the proportion of “*How*” searches using a leave one out analysis. To account for a simple temporal trend, we first removed the linear trend from the dependent variable (“*How*” questions) and the predictor variable (stress levels). The detrended predictor variables were then Z-scored before being entered in the simple linear model. The simple model was then run on all the data saved for one time point which was held out from the analysis. We then used the regression beta to predict the proportion of “*How*” searches of the left-out time point. This process was repeated so that each week’s proportion of “*How*” searches was estimated from the simple model parameters generated without using that week to fit the data. The actual proportion of “*How*” searches of a week (data) and the predicted proportion of “*How*” searches (estimation) were then correlated and also compared using a paired sample *t*-test (see Methods for details). We observed a correlation between the predicted proportion of “*How*” searches (estimate) and the actual proportion of “*How*” searches (data) (r(50) = 0.366, p = 0.007). The means of the two sets of values were not significantly different from one another (t = − 0.041, p = 0.967). This analysis suggests that stress levels in a population is a good predictor of the proportion of “*How*” searches during the pandemic.

Next, we tested whether stress was better predicted by “*How*” Google searches than searches for specific content terms (i.e., “*stress*”, “*anxiety*”, “*mental* *health*” and “*psychiatrist*”), which are often used in attempt to predict population mental state. Thus, we ran multiple linear models to predict stress from each term separately. Once again, the dependent and predictor variables were first detrended and then Z-scored. The strongest association was seen by “*How*” question volume and self-reported stress scores (β = 0.439 ± 0.127 (SE), t(50) = 3.435, p = 0.001, R2 = 0.437), followed by the Google Search Volume Index “*stress*” (β = 0.322 ± 0.134 (SE), t(50) = 2.403, p = 0.020, R2 = 0.322) and “*psychiatrist*” (β = − 0.348 ± 0.131 (SE), t(50) = − 2.887, p = 0.006, R2 = − 0.378); all other predictors were not significant (p’s > = 0.594).

We repeated the above analysis using the average Google Search Volume Index for “*How* *to*” and “*How* *do*,” which may be an even more explicit measure of action-related searches. Our findings remained the same (see Supplementary Analysis).

In the US, we did not have access to measurements of population stress levels. However, we did have access to COVID-19 related confinement data which enabled us to examine the relationship between web searches and residential confinement in the US, when stress levels are not controlled for. We observed that increased COVID-19 related confinement was related to greater Google Search Volume Index “*How*” (β = 0.384 ± 0.129 (SE), t(50) = 2.940, p = 0.005, R2 = 0.384) and to more negatively valenced searches (β = − 0.316 ± 0.134 (SE), t(50) = − 2.354, p = 0.023, R2 = − 0.316). We did not observe a relationship between COVID-19 related confinement and the average Google Search Volume Index of the other question-words (β = − 0.142 ± 0.140 (SE), t(50) = − 1.015, p = 0.315, R2 = − 0.142). Note, all results presented above remain when not detrending.

In addition, we conducted a content analysis to explore the topics of commonly queried “*How*” searches before and after the declaration of a “*National* *Emergency*” in the UK and US, using the LIWC lexicon. We observed that queries regarding work and health were more frequent after a “*National* *Emergency*” was declared relative to before in the UK and US (see Supplementary Analysis).

### Study 3

#### Personal stressful events are associated with an increase in “*How*” and negative valenced searches

To assess whether stressful events influence the propensity to ask “*How*” and the valence of questions in other situations, we ran a third study. First, we asked participants to recall in detail, and write about, either a stressful past event (e.g., “*I* *had* *a* *deadline* *at* *work* *and* *didn't* *know* *if* *I* *was* *going* *to* *meet* *it.*”; stress condition; n = 99, age = 39.45, SD = 14.74; Females = 78.8%, Males = 19.2%, Other = 2.0%) or a relaxing and happy past event (e.g., “*My* *holiday* *in* *[retracted]* *with* *my* *aunt*, *cousin* *and* *her* *children.* *The* *weather* *was* *great*, *nice* *and* *warm.* *We* *were* *staying* *at* *a* *resort* *on* *the* *beach*”); control condition; n = 93, age = 38.46, SD = 11.91; Females = 76.3%, Males = 22.6%, Other = 1.1%). Participants reported their stress level on a scale ranging from *very* *calm* (− 50) to *very* *stressed* (+ 50) before and after recalling the event (see Methods for details).

**Figure 4:**
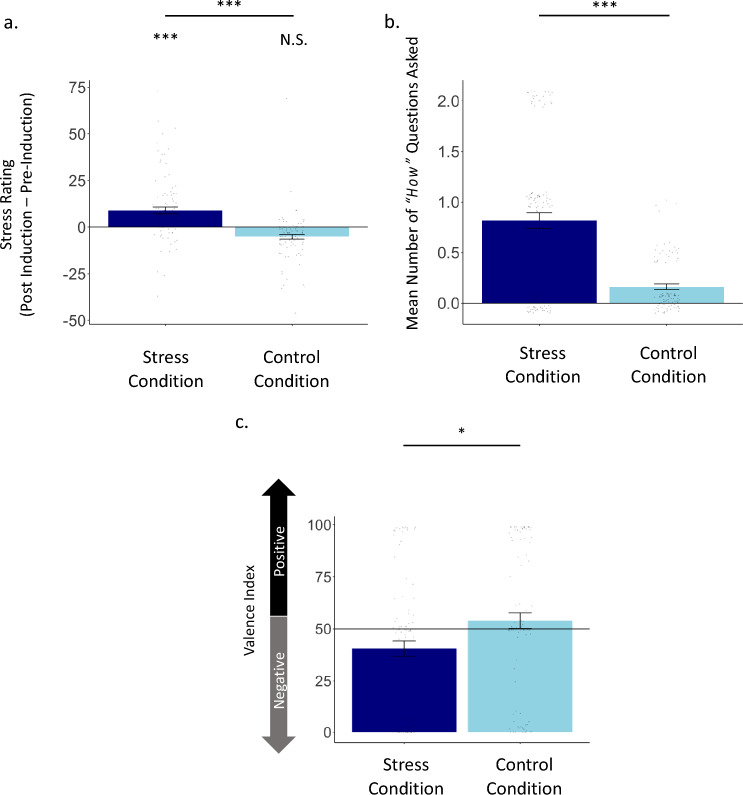
Personal stressful events alter high-level characteristics of searches. (**a**) Participants were asked to recall in detail, and write about, either a stressful past event (i.e., stress condition) or a relaxing past event (i.e., control condition). Participants reported their stress level on a scale ranging from *very* *calm* (− 50) to *very* *stressed* (+ 50) before and after recalling the event. Plotted on the y-axis is participant’s post induction stress rating minus their pre-induction stress rating for the stress condition (dark blue) and control condition (light blue). Participants’ stress scores increased post stress induction compared to pre-induction for the stress condition but not the control condition. (**b**) The mean number of “*How*” questions asked in the stress condition (x-axis; dark blue) was greater than in the control condition (x-axis; light blue). (**c**) In the stress condition (x-axis; dark blue) participants asked more negative questions than in the control condition (x-axis; light blue). Individual scores are shown as dots. Error bars = standard error (SEM). ***p < 0.001, *p < 0.05, *N.S.* not significant (two-sided).

A 2 (condition: stress, control) by 2 (time: pre-induction, post induction) ANOVA on self-reported stress revealed a significant interaction (F(1, 190) = 42.074, p < 0.001, partial eta squared = 0.181). Follow up *t*-tests revealed that the interaction was characterised by participants in the stress condition reporting higher stress post induction (M = − 3.59, SD = 24.30) compared to pre-induction (M = − 12.57, SD = 24.94, *t*(98) = 5.158, p < 0.001, Cohen’s d = 0.518), in contrast, in the control condition, participants mood significantly increased post induction compared to pre-induction (Post induction: M = − 18.80, SD = 27.34, pre-induction: M = − 13.38, SD = 27.79, *t*(92) = − 4.028, p < 0.001, Cohen’s d = − 0.418). Importantly, participants in the stress condition reported greater stress post induction relative to controls (Stress condition: M = − 3.59, SD = 24.30; Control condition: M = − 18.80, SD = 27.34, *t*(190) = 2.581, p < 0.001, Cohen’s d = 0.589), with no difference pre-induction (Stress condition: M = − 12.57, SD = 24.94; Control condition: M = − 13.38, SD = 27.79, *t*(190) = 1.712, p = 0.832, Cohen’s d = 0.031; see Fig. 4a).

Next, participants were asked to enter two searches they could have entered to Google during the time. Our hypothesis is that a stressful event leads to “*How*” searches as an adaptive mechanism to deal with the event, either by acquiring information related to the event directly and/or as a distraction. For each participant, we counted the number of questions that began with “*How*” (i.e., 0, 1, 2) and also assessed the average valence of participants’ two searches, by applying the pre-trained model distilbert-base-uncased-finetuned-sst-2-english^36^. Results show that participants in the stress condition asked significantly more “*How*” questions (M = 0.82, SD = 0.77) than those in the control condition (M = 0.16, SD = 0.28, t(124.58) = 94.154, p < 0.001; Cohen’s d = 1.110; see Fig. 4b). In addition, participants in the stress condition also submitted significantly more negative searches (M = 40.55, SD = 36.51) than those in the control condition (M = 53.97, SD = 36.11, t(190) = − 2.558, p = 0.011; Cohen’s d = − 0.369; see Fig. 4c). This study strengthens the conclusion of Study 2, that stress inducing events are associated with asking more “*How*” questions and negatively valenced question by generalising the finding of Study 2 to other stressful events and providing support for the conclusion in a controlled setting. As shown in Table 1, there was no increase in the use of other question words under stress relative to control.

**Table 1:** An exploratory analysis showed that there was no increase in the use of any other question words under stress relative to control. The use of “*What*” showed a decrease under stress relative to control.

Question type	Mean difference (stress − control)	*t*-test stats
*What*	− 0.43	*t*(144.65) = − 5.179, p < 0.001
*When*	− 0.03	*t*(152.34) = − 1.221, p = 0.224
*Which*	0.01	*t*(190) = 0.969, p = 0.334
*Why*	0.01	*t*(190) = 0.244, p = 0.807
*Where*	− 0.08	*t*(141.80) = − 1.855, p = 0.066
*Who*	− 0.02	*t*(143.47) = − 1.055, p = 0.293

To examine if the increased proportion of asking “*How*” questions in the stress condition was associated specifically with stress rather than with the novelty of the stressful events, we assessed the novelty of events recalled by participants and repeated the analysis above controlling for novelty scores. All results remain the same (see Supplementary Analysis).

In addition, we assessed the semantic similarity between participants’ queries and their recalled events. The analysis revealed a moderate to high level of similarity (see Supplementary Analysis), which suggests that some of the participants questions shared semantic similarity with their recalled event.

## Discussion

The global pandemic generated a new set of practical and mental challenges. To overcome some of these challenges, people turned to technology. On average, people spent almost 7-h a day online in 2020, up 7.3% from the previous year^37^. A large fraction of this time was dedicated to searching for and consuming information^37^. Here, we examined how the high-level features of searches submitted to Google changed in response to the pandemic and private aversive life events and how such changes relate to stress.

In particular, we were interested if under stress people would seek more information that could guide their actions—a behaviour that could facilitate the process of adapting to a stressful event (according to the APA a stressful event can be an occurrence or circumstance that individuals perceive as threatening, challenging, or demanding^38^). Study 1 revealed that participants specifically use the word “*How*” when they want information to guide their action. Thus, in Study 2 we examined the frequency of Google searches that included the word “*How*”. We found that both in the UK and US, the proportion of searches that included the word “*How*” were greater during the year following the declaration of “National Emergency” than in the years prior. It is important to emphasize that any change in the proportion of “*How*” searches cannot simply be explained by a general increase in number of Google searches, as the former are calculated as proportion of the latter. Neither can it be explained by temporal linear trends, as the data was detrended. The rise in “*How*” searches may reflect an adaptive human tendency to ask questions that can facilitate rapid adjustment to new and potentially aversive environments. This interpretation is in accord with a recent study suggesting that changes to search during the pandemic reflect a change in people’s needs^31^. We were also interested in changes to the valence of Google searches during the pandemic relative to before. We observed that the most popular searches in the UK and US were more negatively valenced during the year following the declaration of “*National* *Emergency*” than in the years prior. This aligns with the notion that people may search for information that aligns with their emotional state.

Weekly fluctuations in the proportion of “*How*” questions submitted to Google during the pandemic was positively associated with weekly fluctuations in the proportion of individuals who reported experiencing COVID-related stress in the UK in a sample of over 17K residences. This association could not be attributed to COVID-related confinement, as this factor was controlled for in the model. Furthermore, the relationship was specific to “*How*” searches, and did not generalize to general question asking.

Markedly, we show that the frequency of “*How*” searches predicted COVID-related stress better than the frequency of searches that include stress related content (i.e., “*anxiety*”, “*stress*”, “*mental* *health*”, and “*psychiatrist*”).

Consistent with the above, in Study 3 we show that people are also more likely to ask “*How*” questions, and more negatively valenced questions, in response to a personal stressful life event compared to a non-stressful event. In particular, participants were instructed to recall in detail a stressful life event or a calming event. The former, but not the latter, increased self-reported stress. They were then asked which questions they could have submitted to a search engine at the time. Recalling stressful events was associated with significantly more “*How*” questions and negative questions than control events. This suggests that the relationship between asking “*How*” and stress is not specific to the pandemic or other national events such as national elections but extends to stressful events in general.

This raises the novel idea that tracking the frequency of “*How*” searches may provide clues regarding population-level stress beyond the time of a pandemic and perhaps provide clues regarding an individual’s stress level. Of course, while we show (in Study 3) that stress causes a rise in “*How*” questions, a rise in “*How*” questions does not necessitate a rise in stress, as there may be other reasons for why people ask more “*How*” questions. Thus, tracking “*How*” queries cannot on its own be an indicator of stress but may provide an indicator of possible rising stress levels which can be then followed up using other indicators.

Our investigation was guided by previously identified factors that motivate information-seeking (for review see^8^). In particular, studies show that people seek information more when it is useful in guiding action^9,39–41^. The current results suggest that this motive is ‘up weighted’ when experiencing stress, perhaps because the need to select adaptive actions is heightened under such circumstances. While past studies also show people prefer to seek good news over bad^8,39,41–48^ we find that under stress web searches were in fact more negative. This could simply be due to a significant proportion of searches relating to the stress itself (i.e., illness in Study 2 and a range of personal aversive events in Study 3). We did not, however, observe a significant association between valence of web searches and self-reported stress levels. It is interesting to note that studies examining information-*sharing* (e.g., tweets) rather than information-*seeking* have revealed small but significant associations between mental state and the valence of information *shared*^49–51^. Because a significantly smaller slice of the population regularly shares than seeks information, understanding how information-seeking relates to mental health is crucial and may diverge from patterns observed for information *sharing*.

Reflecting on why individuals tend to ask action-oriented questions during stressful times is crucial. Our theory posits that asking “*How*” questions could offer a sense of control, potentially mitigating stress by empowering individuals with knowledge and actionable strategies. This actionable information may be directly relevant to dealing with the stressor (e.g., if a hurricane is coming one may ask “*how* *do* *I* *duck-tape* *windows*?”) or aimed at actions that can distract and reduce stress (e.g., if a hurricane is coming one may ask “*how* *do* *you* *play* *scrabble*?”). This hypothesis aligns with the notion that acquiring control, or even the perception of it, can be a key factor in stress alleviation.

Together, the findings show that in the face of high stress people search for information that can help guide action. While in the past such information may have been sought directly from other people, with the development of the internet, individuals are now able to turn to the web for answers. This ability may have contributed to the high resilience and quick adaptation observed in response to the pandemic^6,52,53^.

## Materials and methods

### Study 1

#### Participants

One-hundred participants (Age = 39.29 (SD = 13.86), Females = 56%, Males = 44%, Other = 0%) completed the study on Qualtrics (http://www.qualtrics.com) and were recruited via Prolific’s online recruitment system (http://www.prolific.co). Participants received £7.50 per hour for their participation. For all studies presented in this article, ethical approval has been provided by the UCL Ethics Committee [12467/005 and 3990/003] and all participants have given their informed consent to participate. All methods were performed in accordance with UCL’s guidelines and regulations.

#### Procedure

Participants were asked to think about a goal they were trying to achieve. They were then instructed to select from a list of eight question-words (“*What*”, “*Which*”, “*Who*”, “*Where*”, “*Why*”, “*When*”, “*Whose*”, and “*How*”) a word that they would use to submit a query on Google to help guide their actions to achieve their goal (i.e., the test question). Participants were also asked to think about a topic they are interested in learning more about and select a question-word from the same list to ask a query on Google to increase their knowledge about that topic (i.e., the control question). The order of the two questions were counter balanced, and the order of the question-words were randomized. This design allowed us to identify which words people use to seek information to guide action and test whether such words were used generally for information-seeking and more specifically to guide action.

#### Analysis

To assess which question-words were associated with guiding action, we first calculated the proportion of people that selected each of the question-words. We then conducted a Fisher’s exact test to examine whether the most prevalent question-word selected (i.e., “*How*”) was significantly different than the proportion of all other question-words asked together for each question separately (coded ‘0’ if “*How*” was selected and ‘1’ if any other question-word was selected), and whether the proportion of people that selected “*How*” for the test question was significantly different than for the control question.

### Study 2

#### Data extraction

##### Web search data

Weekly search data was extracted from Google Trends (http://www.googletrends.com) for 220 weeks (January 1st, 2017 through March 21st, 2021). This was done separately for the UK and the US. Based on the results of Study 1, to quantify action guidance, we extracted the Google Search Volume Index for the search term “*How*”*.* We also extracted the Google Search Volume Index for the search terms “*What*”, “*Which*”, “*Who*”, “*Where*”, “*Why*”, “*When*”, and “*Whose*” and then averaged them together to quantify general question asking (i.e., control variable). A Google Search Volume Index value is equal to the number of searches for the specific term of interest in a given week and region (for example total number of searches that include the question-word “*How*” in the UK the first week of 2020) divided by the total number of searches in that same time and region (for example the total number of Google searches submitted in UK the first week of 2020). These values are normalized to represent search interest relative to the highest value for that region for the entire time frame (i.e., January 1st, 2017–March 21st, 2021).

To quantify valence, we extracted the 25 most popular searches for each week and region for “*What*”, “*Which*”, “*Who*”, “*Where*”, “*Why*”, “*When*”, “*Whose*” and “*How*”, questions. That is, for each week we extract up to 25 searches per type of question (i.e., ~ 200 total web searches for each week), as this is the maximum Google Trends reports. Next, we implemented a machine learning approach to assess the valence of these searches, by applying the pre-trained model distilbert-base-uncased-finetuned-sst-2-english^36^ to the extracted data. The models output contains two labels: positive and negative, along with a score between 1 and 0 (1 = absolute confidence in the model output label, 0 = zero confidence in the model output label). The sum of the values is always equal to 1, for example, a potential output could be Positive = 0.9, Negative = 0.1. As the two measures are fully dependent, we used the positive confidence score as our measurement of valence and then transformed this number to be on a scale from 0 to 100, such that it would be easily comparable to the Google Search Volume Index for “*How*”, described above (i.e., a score of 100 denotes the most positively valenced score and a score of 0 the most negatively valenced score).

##### Self-reported stress

Data was extracted, with permission, from the UK COVID-19 Social Study^32^. The study is a panel study of over 70,000 UK citizens which aims to characterize the psychological and social experience of adults living in the UK during the COVID-19 pandemic (see Table 2 for demographics). The study commenced as a weekly survey, with participants receiving an invitation to the next wave of data collection 7 days following their last completion. All participants received up to 2 reminders (24 and 48 h following their initial weekly invitation). The link to their last reminder remained live so they could return to the study a few days later if they chose to. Following week 22 of the study, monthly follow-ups rather than weekly follow-ups were sent. To attain an equal number of responses across time, participants were randomized to receive their monthly invitation on either week 1, 2, 3 or 4 of the month, with subsequent invitations following 28 days after they completed the survey. An average of 17,468 individuals submitted data each week (see Table 3 for response frequency for each week). For full methods and demographics for the sample see http://www.COVIDSocialStudy.org. The UK COVID-19 Social Study was approved by the UCL Research Ethics Committee [12467/005 and 3990/003], and all participants gave written informed consent. All methods were performed in accordance with UCL’s guidelines and regulations.

**Table 2:** Demographics of respondents in the UK COVID-19 Social Study (adapted from Fancourt and colleagues^32^). Data in the Fancourt and colleagues^32^ study and in our study are weighted using auxiliary weights to the national census and Office for National Statistics (ONS) data.

	Number of observations	%		Number of observations	%
Age			Education levels		
18–29	51,858	5.77	GCSE or below	126,427	14.1
30–59	493,016	54.9	A-levels of equivalent	154,954	17.3
60+	353,559	39.4	Degree or above	617,052	68.7
Gender			Any diagnosed mental health conditions		
Male	225,578	25.2	No	748,416	83.3
Female	669,279	74.8	Yes	150,017	16.7
Ethnicity			Any diagnosed physical health conditions		
White	860,157	96.0	No	516,884	57.5
Ethnic minority	35,455	3.96	Yes	381,549	42.5
UK nations			Keyworker		
England	725,705	81.6	No	711,201	79.2
Wales	108,598	12.2	Yes	187,232	20.8
Scotland	55,416	6.23	Living with children		
Living arrangement			No (excluding those who live alone)	510,650	72.0
Not living alone	709,289	79.0	Yes	198,639	28.0
Living alone	189,144	21.1	Living area		
Annual household income			Village/hamlet/isolated dwelling	225,022	25.1
> 30k	482,268	59.6	City/large town/small town	673,411	75.0
< 30k	327,187	40.4

**Table 3:** The total number of participants providing data during each calendar week in the UK COVID-19 Social Study (adapted from Fancourt and colleagues^32^).

Date	Week	Freq	Date	Week	Freq
21/03/20–27/03/20	1	28,929	19/09/20**–**25/09/20	27	8,318
28/03/20–03/04/20	2	27,873	26/09/20**–**02/10/20	28	8,366
04/04/20–10/04/20	3	38,151	03/10/20**–**09/10/20	29	8,501
11/04/20–17/04/20	4	38,453	10/10/20**–**16/10/20	30	8,072
18/04/20–24/04/20	5	38,504	17/10/20**–**23/10/20	31	7,495
25/04/20–01/05/20	6	36,513	24/10/20**–**30/10/20	32	7,612
02/05/20–08/05/20	7	36,651	31/10/20**–**06/11/20	33	7,830
09/05/20–15/05/20	8	37,549	07/11/20**–**13/11/20	34	7,443
16/05/20–22/05/20	9	35,702	14/11/20**–**20/11/20	35	6,995
23/05/20–29/05/20	10	33,293	21/11/20**–**27/11/20	36	7,078
30/05/20–05/06/20	11	32,196	28/11/20**–**04/12/20	37	7,190
06/06/20–12/06/20	12	31,304	05/12/20**–**11/12/20	38	6,947
13/06/20–19/06/20	13	30,229	12/12/20**–**18/12/20	39	6,473
20/06/20–26/06/20	14	29,153	19/12/20**–**25/12/20	40	6,240
27/06/20–03/07/20	15	28,534	26/12/20**–**01/01/21	41	6,966
04/07/20–10/07/20	16	27,552	02/01/21**–**08/01/21	42	7,038
11/07/20–17/07/20	17	26,737	09/01/21**–**15/01/21	43	6,274
18/07/20–24/07/20	18	25,983	16/01/21**–**15/01/21	44	6,219
25/07/20–31/07/20	19	25,005	23/01/21**–**29/01/21	45	6,540
01/08/20–07/08/20	20	24,530	30/01/21**–**05/02/21	46	6,831
08/08/20–14/08/20	21	23,851	06/02/21**–**12/02/21	47	6,048
15/08/20–21/08/20	22	23,120	13/02/21**–**19/02/21	48	6,217
22/08/20–28/08/20	23	11,373	20/02/21**–**26/02/21	49	6,111
29/08/20–04/09/20	24	10,025	27/02/21**–**05/03/21	50	6,574
05/09/20–11/09/20	25	9,916	06/03/21**–**12/03/21	51	8,683
12/09/20–18/09/20	26	10,009	13/03/21**–**19/03/21	52	9,128

Participants were asked: “*over* *the* *past* *week*, *have* *any* *of* *the* *following* *been* *worrying* *you* *at* *all*, *even* *if* *only* *in* *a* *minor* *way*?” They were presented with 18 factors that may cause worry (for example internet access, boredom, neighbours) and were to pick any that they were worried about. Five of these factors were a-priori categorized by the authors of the survey^32^ as ones that have been impacted by COVID. These were (i) catching COVID-19 (ii) becoming seriously ill from COVID-19, (iii) finances, (iv) losing your job/unemployment and (v) getting food. Second, they were asked “*have* *any* *of* *these* *things* *been* *causing* *you* *significant* *stress?* (*e.g.*, *they* *have* *been* *constantly* *on* *your* *mind* *or* *have* *been* *keeping* *you* *awake* *at* *night*)”. They were presented with the same 18 factors as above and were asked to tick any of those causing significant stress. For each week and factor, Fancourt and colleagues^32^ calculated the proportion of respondents that ticked that factor either in response to question 1 and/or question 2. Factors i and ii were a-priori combined by Fancourt and colleagues^32^ to make one factor, leaving us with four factors. For each week the proportion of people ticking 1 and/or 2 were averaged across the four factors to produce one indicator of “stress levels” for that week.

Table 2 shows the demographic of respondents to the UK COVID-19 Social Study. Importantly, data points reported by Fancourt and colleagues^32^ were weighted using auxiliary weights to the national census and Office for National Statistics (ONS) data. We used these weighted data points in our study. Thus, reported stress levels are representative of the UK population.

##### COVID-19 confinement score

To measure COVID-19 related confinement, we extracted eight confinement variables from a publicly available dataset (The Oxford University COVID-19 Government Response Tracker^33^). All variables are ordinal coded by severity/intensity of confinement, on a daily basis (from January 1st, 2020 to March 21st, 2021), for the following: (i) *school* *and* *university* *closures*, (ii) *workplace* *closures*, (iii) *public* *event* *cancelations*, (iv) *restrictions* *on* *gatherings*, (v) *public* *transport* *restrictions*, (vi) *stay* *at* *home* *requirements*, (vii) *restrictions* *on* *domestic* *travel*, and (viii) *restrictions* *on* *international* *travel;* see Table 4 for coding*.* To obtain weekly values, we computed weekly averages of the daily ratings. To quantify an overall COVID-19 related confinement score, we transformed all variables to range between 0 and 1 using the R function *scaler* from the R package, *bruceR*. Finally, we averaged the 8 transformed variables together.

**Table 4:** Coding of COVID-19 related confinement variables (adapted from Hale and colleagues^33^).

Variable description	Coding instructions
*Closings* *of* *schools* *and* *universities*	0—No measures 1—Recommend closing, or all schools open with alterations resulting in significant differences compared to usual, non-COVID-19 operations 2—Require closing (only some levels or categories, e.g., just high school, or just public schools) 3—Require closing all levels
*Closings* *of* *workplaces*	0—No measures 1—recommend closing (or work from home) 2—require closing (or work from home) for some sectors or categories of workers 3—require closing (or work from home) all-but-essential workplaces (E.g., grocery stores, doctors)
*Cancelling* *public* *events*	0—No measures 1—Recommend cancelling 2—Require cancelling
*Cut-off* *size* *for* *bans* *on* *gatherings*	0—No restrictions 1—Restrictions on very large gatherings (the limit is above 1000 people) 2—Restrictions on gatherings between 101–1000 people 3—Restrictions on gatherings between 11–100 people 4—Restrictions on gatherings of 10 people or less
*Closing* *of* *public* *transport*	0—No measures 1—Recommend closing (or significantly reduce volume/route/means of transport available) 2—Require closing (or prohibit most citizens from using it)
*Orders* *to* “*shelter-in-* *place*” *and* *otherwise* *confine* *to* *home*	0—No measures 1—recommend not leaving house 2—require not leaving house with exceptions for daily exercise, grocery shopping, and ‘essential’ trips 3—Require not leaving house with minimal exceptions (E.g., allowed to leave only once a week, or only one person can leave at a time, etc.)
*Restrictions* *on* *internal* *movement*	0—No measures 1—Recommend not to travel between regions/cities 2—internal movement restrictions in place
*Restrictions* *on* *international* *travel*	0—No measures 1—Screening 2—Quarantine arrivals from high-risk regions 3—Ban on arrivals from some regions 4—Ban on all regions or total border closure

### Analysis

Analysis was conducted separately for data from the UK and the US. In each region we quantified action guidance searches (i.e., “*How*”) and valence of searches every week from the date the “*National* *Emergency*” was declared due to the COVID-19 pandemic, (March 23rd, 2020 in the UK and March 13th, 2020 in the US) through March 21st, 2021, as well as every week dating back to January 1st, 2017. We then compared the weekly scores before the “*National* *Emergency*” to that after using an independent samples *t*-test.

To assess whether our measures were related to UK stress levels, we conducted two linear models predicting on a weekly basis Google’s Search Volume Index of “*How*” questions and the Valence Index of questions submitted to Google in the UK, from UK stress levels. We also included weekly COVID-related confinement scores in the models to disentangle the effects of stress from the effects of confinement due to restrictions placed by the Government. To account for simple temporal trends, we removed the linear trend from the dependent and predictor variables first, using the *detrend* function in the *pracm*a R package. The detrended dependent and predictor variables were then Z-scored.

Next, to examine whether the relationship between stress and “*How*” searches was specific or rather reflected a general tendency to ask more questions when stressed we conducted a third linear model relating question asking to stress levels controlling for COVID-related confinement scores. To do so, we extracted the Google Search Volume Index for all other common question-words (i.e., “*What*”, “*Which*”, “*Who*”, “*Where*”, “*Why*”, “*When*”, and “*Whose*”; Z-scored and detrended) and then averaged them together.

We were then interested in whether stress was better predicted by “*How*” Google searches than searches for stress related terms. To test this, we first removed the linear trend from the dependent and predictor variables, and then Z-scored the dependent and predictor variables. We then ran a model predicting the proportion of UK sample reporting COVID-related stress from the “*How*” Google Search Volume Index as well as Google Search Volume Index for the words “*stress*”, “*anxiety*”, “*mental* *health*”, and “*psychiatrist*” in the UK. In addition, we ran multiple linear models to predict UK COVID-related stress levels each time from only one of the terms above.

Finally, we tested the predictive validity of a simple model using stress levels to predict the proportion of “*How*” searches using a leave one out analysis. Once again, we removed the linear trend from the dependent and predictor variables first, and then the dependent and predictor variables were Z-scored. Specifically, the simple model was run on all the data saved for one time point which was held out from the analysis. We then used the regression beta to predict the proportion of “*How*” searches of the left-out time point. This process was repeated so that each week’s proportion of “*How*” searches was estimated from the simple model parameters generated without using that week to fit the data. This resulted in two values for the proportion of “*How*” searches for each week: the actual proportion of “*How*” searches (data) and the predicted value from the leave-one-out validation (estimate). The actual proportion of “*How*” searches of a week (data) and the predicted proportion of “*How*” searches (estimation) were then correlated and compared using a paired sample *t*-test. This analysis indicates whether the population stress levels is a good predictor of the proportion of “*How*” searches.

### Study 3

#### Participants

One hundred and ninety-three participants completed the study on Qualtrics (http://www.qualtrics.com) and were recruited via Prolific’s online recruitment (http://www.prolific.co). Participants received £7.50 per hour for their participation. One participant was excluded for not providing a valid response, leaving the final participant N at 192 (stress condition: n = 99, age = 39.45, SD = 14.74; Females = 78.8%, Males = 19.2%, Other—2.0%; control condition: n = 93, age = 38.46, SD = 11.91; Females = 76.3%, Males = 22.6%, Other = 1.1%).

#### Procedure

Participants were asked to recall a time when they were very stressed (*stress* *condition*) or recall a time when they were happy and relaxed (*control* *condition*). They were then instructed to think about that time in as much detail as possible and describe it in a text box. They indicate their stress level on a scale ranging from very calm (− 50) to very stressed (+ 50) before and after the induction. Next, participants were asked to enter two searches they could have entered to Google during the time.

#### Analysis

To assess whether the manipulation was successful, a 2 (condition: stress, control) by 2 (time: pre-induction, post induction) ANOVA was run with follow up pair-wise *t*-tests. Next, for each participant, we counted the number of questions that began with “*How*” (i.e., 0, 1, 2) and for every other question word (i.e., “*What*”, “*Which*”, “*Who*”, “*Where*”, “*Why*”, “*When*”, and “*Whose*”). We then conducted separate independent samples *t*-tests to assess the difference in the number asked for each question type between conditions. Finally, we implemented a machine learning approach to assess the average valence of participants’ two searches by applying the pre-trained model distilbert-base-uncased-finetuned-sst-2-english^36^ to their submitted searches. Finally, we examined the difference between participants in the stress and control conditions with regard to the number of “*How*” searches submitted and the valence of their searches.

### Supplementary Information

Supplementary Information.

## Data Availability

Data and code are available at a dedicated OSF repository (https://osf.io/edr74/?view_only=f2c80dcafd2447e5a4a06567cfa26b2a). The source data underlying Figs. 1, 2, 3, 4 are provided as part of this repository.

## Code availability

Code supporting this study are available at a dedicated OSF repository (https://osf.io/edr74/?view_only=f2c80dcafd2447e5a4a06567cfa26b2a).
