# Supplementary material for: “How” web searches change under stress
Source: Sci Rep. 2024 Jul 2;14:15147. doi: 10.1038/s41598-024-65895-4 (PMC11220009; doi:10.1038/s41598-024-65895-4)
Supplement: Supplementary file 1 — Supplementary Information. [file 41598_2024_65895_MOESM1_ESM.docx]

**Supplementary**

**Replication of Study 2 findings using the Mean of Google Search Volume Index of *“How to” & “How do”*.**

We tested whether the findings of Study 2 held true when using the average Google Search Volume Index for “*How to*” and “*How do*”, which may be an even more explicit measure of action-related searches. First, in the UK and US we observed a significant increase in the average Google Search Volume Indexes of *“How to” and “How do”* questions following the declaration of a *“National Emergency”* relative to the three years previous (UK Mean *“How to” & “How do”*: before *“National Emergency”* declared: M = 61.63, SD = 2.46, after *“National Emergency”* declared: M = 79.83, SD = 8.49, t(53.67) = -15.257, p < 0.001, Cohen’s d = 4.636; US Mean *“How to” & “How do”*: before *“National Emergency”* declared: M = 63.67, SD = 1.72, after *“National Emergency”* declared: M = 76.68, SD = 5.48, t(55.28) = -17.024, p < 0.001, Cohen’s d = 3.067).

Next, a linear model predicting the UK Mean of *“How to” & “How do”* questions from stress levels and COVID-19 related confinement scores, revealed that both high stress (β = 0.227 ± 0.081 (SE), t(51) = 2.783, p = 0.008) and greater COVID-19 related confinement (β = 0.771 ± 0.075 (SE), t(51) = 10.234, p < 0.001) predicted proportion of *“How to” & “How do”* searches.

Finally, we tested whether stress was better predicted by the UK Mean of *“How to” & “How do”* Google searches than searches for specific content terms (i.e., *“stress”*, *“anxiety”, “mental health”* and *“psychiatrist”*), which are often used in attempt to predict population mental state. Thus, we ran multiple linear models to predict stress from each term separately. Once again, the dependent and predictor variables were first detrended and then Z-scored. The strongest association was seen between the UK Mean of *“How to” & “How do”* question volume and self-reported stress scores (β = 0.460 ± 0.126 (SE), t(50) = 3.661, p < 0.001, R2= 0.460), followed by the Google Search Volume Index for “*stress*” (β = 0.322 ± 0.134 (SE), t(50) = 2.403, p = 0.020, R2= 0.322) and *“psychiatrist”* (β = -0.348 ± 0.131 (SE), t(50) = -2.887, p = 0.006, R2= -0.378); all other predictors were not significant (p’s >= 0.594).

**Findings from Study 3 remain after controlling for novelty.**

To assess whether novelty rather than stress was driving the results, we asked 25 new participants to rate the events submitted by the original participants on a 6-point Likert scale from “*not at all novel*” to “*very much* novel”. Participants were instructed that a "*very much novel*” event is one that is probably quite rare or entirely new to the person experiencing it, while a "*not at all novel*" event is one that is likely very commonplace or familiar.

First, we examined if the stress manipulation was successful. Indeed, a linear model predicting the change in subjective stress rating after the manipulation relative to before from condition (0 = Stress condition, 1 = Control condition) while accounting for novelty, revealed that the increase in stress was greater in the stress manipulation group relative to control group, (β = -14.243 ± 2.245 (SE), t(189) = -6.344, p < 0.001). Novelty of the event, on the other hand, was not predictive of stress change (β = -0.892 ± 0.430 (SE), t(189) = -3.120, p = 0.430).

Importantly, “*How*” searches were more frequent in the stress manipulation group than the control group, while accounting for novelty (β = -1.015 ± 0.126 (SE), t(189) = -8.029, p < 0.001). Novelty ratings were also associated with more “*How*” questions (β = -0.172 ± 0.064 (SE), t(189) = -2.712, p = 0.007).

Moreover, Valence Index of queries in the stress manipulation group were more negative than queries of the control group, while accounting for novelty (β = 0.333 ± 0.144 (SE), t(189) = 2.315, p = 0.022). Novelty was not associated with query valence (β = -0.070 ± 0.072 (SE), t(189) = -0.970, p = 0.333). Together, these analyses suggest that even when novelty is accounted for, those in the stress condition ask more “*How*” queries and ask more negative queries compared to those in the control group.

**The propensity of “*How*” queries predict stress levels better than the specific topic of queries.**

We conducted an additional analysis to explore the composition of commonly queried *“How”* searches before and after the declaration of a “*National Emergency*” in the UK and US. We computed the mean percentage of words extracted from the top 25 most popular “*How*” searches each week before and after a “*National Emergency*” was declared in the UK and US. These words were categorised based on the General Topics of the LIWC lexicon, including Culture (with subgroups: politics, ethnicity, tech), Lifestyle (with subgroups: leisure, home, work, money, religion), and Physical (with subgroups: health - including illness, wellness, mental health - substances, sexual, food, death). Note that, like the valence analysis mentioned in the manuscript, only the top 25 search queries each week are available for extraction for a specific search term (i.e., “*How*”). The bar chart below illustrates the mean percentage of words extracted from the top 25 most popular “*How*” searches each week before (grey) and after (purple) a *National Emergency* declaration, categorised by LIWC General Topics. Significance testing via independent sample t-tests was conducted to compare the periods before and after the “*National Emergency*”. We observed that the only topics that consistently changed after a “*National Emergency*” was declared compared to before in the UK and US was work and health. This is not surprising as they are two topics which were impacted considerably with the pandemic.

**Supplementary Figure.** Comparison of Mean Percentage of Words from Top 25 “*How*” Searches Before and After National Emergencies in the **(a)** UK and **(b)** US, Categorized by LIWC Lexicon General Topics. Grey bars represent the mean percentage of words before the *National Emergency* declaration, while purple bars represent the mean percentage after. The *National Emergency* was assessed from March 23^rd^, 2020 to March 21^st^, 2021, in the **(a)** UK and from Match 13^th^, 2020 to March 21^st^, 2021 in the **(b)** US. Error bars indicate standard error of the mean (SEM), and significance stars denote p-values from independent sample t-tests comparing before and after the declaration of a *National Emergency* (*p < 0.05, ** p < 0.01, *** p < 0.001).

Next, we ran multiple linear models to predict stress from each LIWC General Topic category separately. We also predicted UK population stress levels from the UK Google Search Volume Index for “*How*” as a comparison. Once again, the dependent and predictor variables were first detrended and then Z-scored. The results revealed that the increased proportion of asking “*How*” predicted UK population stress levels (β = 0.436 ± 0.172 (SE), t(49) = 3.435, p < 0.001), while none of the LIWC General Topic variables were significant (all p-values > 0.096). This suggests that using the Google Search Volume Index may be a better indicator of stress levels than quantifying specific topics from the top 25 searches each week.

**Similarity between participants' queries and recalled events.**

To investigate the relationship between participants' queries and their recalled events in Study 3, we employed a semantic similarity assessment utilising Bidirectional Encoder Representations from Transformers (BERT). To do so, we leveraged BERT's pre-trained 'bert-base-uncased' model to generate embeddings for the participants' responses. This model, developed by Devlin and colleagues (2018), provides state-of-the-art word representations that capture a wide range of syntactic and semantic information. Then for each participant, cosine similarity scores were calculated between the BERT-generated embeddings of their queries and the embeddings of their recalled events. Cosine similarity measures the cosine of the angle between two vectors, offering a metric for the semantic proximity of text data, with a score of 1 indicating perfect similarity. Finally, using the derived similarity scores, we performed statistical analyses to quantify the descriptive statistics for each.

The analysis of similarity scores between participants’ queries and their recalled events revealed comparable means across both Stress and Control conditions, with 0.55 (SD = 0.09) and 0.54 (SD = 0.08) respectively, indicating a moderate to high level of similarity.
